# Optimising Olive Leaf Phenolic Compounds: Cultivar and Temporal Interactions

**DOI:** 10.3390/plants14172789

**Published:** 2025-09-05

**Authors:** Igor Pasković, Mario Franić, Theocharis Chatzistathis, Paula Pongrac, Paula Žurga, Valerija Majetić Germek, Igor Palčić, Smiljana Goreta Ban, Mariem Zakraoui, Šime Marcelić, Jure Mravlje, Joško Kaliterna, Marija Polić Pasković

**Affiliations:** 1 Department of Agriculture and Nutrition, Institute of Agriculture and Tourism, Karla Huguesa 8, 52440 Poreč, Croatia; paskovic@iptpo.hr (I.P.); mario@iptpo.hr (M.F.); palcic@iptpo.hr (I.P.); smilja@iptpo.hr (S.G.B.); 2 Faculty of Health Studies, University of Rijeka, Viktora Cara Emina 5, 51000 Rijeka, Croatia; 3 Institute of Soil and Water Resources, Hellenic Agricultural Organisation (ELGO) ‘DIMITRA’, 57001 Thessaloniki (Thermi), Greece; 4 Jožef Stefan Institute, Jamova 39, 1000 Ljubljana, Slovenia; paula.pongrac@bf.uni-lj.si (P.P.); jure.mravlje@bf.uni-lj.si (J.M.); 5 Department of Biology, Biotechnical Faculty, University of Ljubljana, Jamnikarjeva 111, 1000 Ljubljana, Slovenia; 6 Teaching Institute of Public Health of Primorsko-Goranska County, Krešimirova 52a, 51000 Rijeka, Croatia; paula.zurga@zzjzpgz.hr; 7 Department of Food Technology and Control, Faculty of Medicine, University of Rijeka, Brace Branchetta 20, 51000 Rijeka, Croatia; valerija.majetic@medri.uniri.hr; 8 Faculty of Sciences of Tunis, University of El Manar, Tunis 2092, Tunisia; mariem.zakraoui@gmail.com; 9 Department for Ecology, Agronomy and Aquaculture, University of Zadar, Trg Kneza Višeslava 9, 23000 Zadar, Croatia; simemarcelic@unizd.hr; 10 Faculty of Agriculture, University of Zagreb, Svetošimunska cesta 25, 10000 Zagreb, Croatia; jkaliterna@agr.hr

**Keywords:** oleuropein, verbascoside, Rošinjola, Buža muška, biochemical farming strategy

## Abstract

All olive (*Olea europaea* L.) plant tissues have a high phenolic content. However, the effects of the cultivar and sampling period on the tissue phenolic content remain almost unknown; in addition, the interactions between nutrient uptake and leaf phenol concentrations have not been clarified. This study sampled olive leaves to explore how the cultivar, sampling period, and their interaction affect leaf phenol and nutrient concentrations. Leaves were collected from six cultivars during three seasonal periods: harvest (October; SP1), dormancy (January; SP2), and pruning (March; SP3). Five were Istrian cultivars (‘Bova’, ‘Buža muška’, ‘Buža puntoža’, ‘Istarska bjelica’, ‘Rošinjola’), and one was the Italian cultivar ‘Leccino’. Phenolic profiles in olive leaves were correlated with potassium (K), phosphorus (P), and copper (Cu) concentrations. However, significant correlations between these nutrients and oleuropein, verbascoside, and total phenolic content (TPC) were determined only for ‘Rošinjola’. Oleuropein was the most abundant phenolic compound, while among genotypes, ‘Buža muška’ showed the highest oleuropein levels across all sampling periods, indicating its potential source of oleuropein in olive leaves. Seasonal variations in olive leaf phenolic compounds appear to be strongly influenced by phenological phase, nutrient dynamics, and weather conditions, as confirmed by multivariate analysis across sampling periods and cultivars. The findings emphasise the importance of selecting both an appropriate cultivar and sampling period to maximise the accumulation of olive leaf phenolic compounds. Nevertheless, long-term experimentation on cultivars with a high leaf phenolic potential, like ‘Buža muška’ and ‘Rošinjola’, is necessary in order to develop appropriate farming strategies for maximising phenolic compounds with human or plant health benefits.

## 1. Introduction

Olive (*Olea europaea* L.) is the most widespread tree crop in the Mediterranean basin, with a significant economic and environmental role. The Istrian region (traversing Croatia and Slovenia) is an established olive-growing and oil-producing area, traditionally recognised for its autochthonous olive cultivars. Olive oil quality, including secondary metabolite content, is of high importance for the local economy. In contrast, leaves remain less studied, despite their potential as a valuable source for the production of phenolic compounds, antioxidants, and other phytochemicals. Olive cultivation produces substantial quantities of waste biomass; approximately 25 kg of twigs and leaves annually from each tree is removed by pruning, while leaves are estimated to account for approximately 10% of the total harvest weight and are separated from the fruits before the oil extraction process [1].

Olive leaves contain numerous bioactive compounds, most abundant being oleuropein; some of these compounds have been reported to have antimicrobial, hypoglycaemic, anti-hypertensive, and anti-inflammatory properties [2]. Historically, they have been used to treat fever and malaria [3]. In certain regions, dry or fresh olive leaves have been and still are used for making hot beverages, so-called “olive tea”. The concentration and profile of phenolic compounds in olive leaves depend on the genotype, tree age, environmental conditions, agricultural practices, and their interactions, as well as other different biotic and abiotic factors, such as physical injury, infection, heavy metal–salt stress, etc. [4,5,6,7,8]. Among these factors, genotype exerts a particularly decisive role, with phenolic and nutrient profiles proposed as biochemical markers for cultivar identification and geographic origin [8,9]. Additionally, recent research on the topic emphasises that the regulation of phenylpropanoid metabolism and the accumulation of phenolic compounds in various plant species are significantly affected by environmental conditions and phenological stages, as well as their interaction [6,7]. Therefore, the sampling period can be highlighted as an essential factor that reveals general patterns of phenolic compound dynamics across different olive cultivars’ growth and development.

Phenolic compounds also contribute significantly to the olive tree’s defence system, acting as pre-formed antimicrobial agents (phytoanticipins), which are particularly effective against fungal pathogens [10]. In olive trees, foliar application of specific micronutrients such as copper and boron has been shown to increase the phenolic content [11,12] and enhance the mycelial growth inhibition [11]. Furthermore, levels of certain phenolic compounds, such as tyrosol, oleuropein, and verbascoside, have been associated with resistance to major olive pests and pathogens, such as *Xylella fastidiosa*, *Phytophthora megasperma*, *Cylindrocarpon destructans*, and *Spilocaea oleagina* [13,14,15].

Taken together, these findings reinforce the bioprotective role of specific phenolic compounds and emphasise their potential value for phytochemical-oriented breeding and valorisation strategies. A deeper understanding of the genotype-dependent variation in phenolic content, particularly concerning environmental and management factors, could support the targeted selection of cultivars for biostimulant production, natural plant protection, and the extraction of functional ingredients from olive leaves, offering a promising pathway to sustainable disease management.

In addition to the genotypic effect, seasonal variation has been shown to significantly influence secondary metabolites in olive leaves, as well as their biosynthesis, stability, and degradation [4]. Therefore, optimising the phenolic content in olive leaves through the appropriate selection of genotype and harvesting time is crucial for enhancing their value and farmers’ income. Given that there are more than one million olive trees cultivated in the Istrian region [16], it is evident that leaves, as waste material, have a potentially significant commercial value, lost mainly by pruning and burning [17].

The impact of nutrient status on phenolic compound concentrations in plant tissue has been previously reviewed by Treutter et al. [18]. Although it is well established that fertilisation can significantly alter the phenolic profile of plants [19], relatively few studies have focused on the effects of mineral nutrition on the phenolic composition of olive leaves [4]. Moreover, limited attention has been devoted to how nutrient status influences phenolic compounds during distinct phenological stages (harvest, dormancy, pruning) in selected Istrian cultivars.

Therefore, this study aimed to assess the effects of genotype, sampling period, and olive leaf mineral status on the phenolic composition of olive leaves. The specific objectives were:

To examine the correlation between specific olive leaf micronutrients (Cu and B) and key phenolic compounds;

To determine seasonal variation in phenolic concentrations across three sampling periods;

To identify high-phenolic cultivars suitable for the valorisation of olive leaf biomass.

## 2. Results

### 2.1. Leaf Phenolic Content

**Figure 1:**
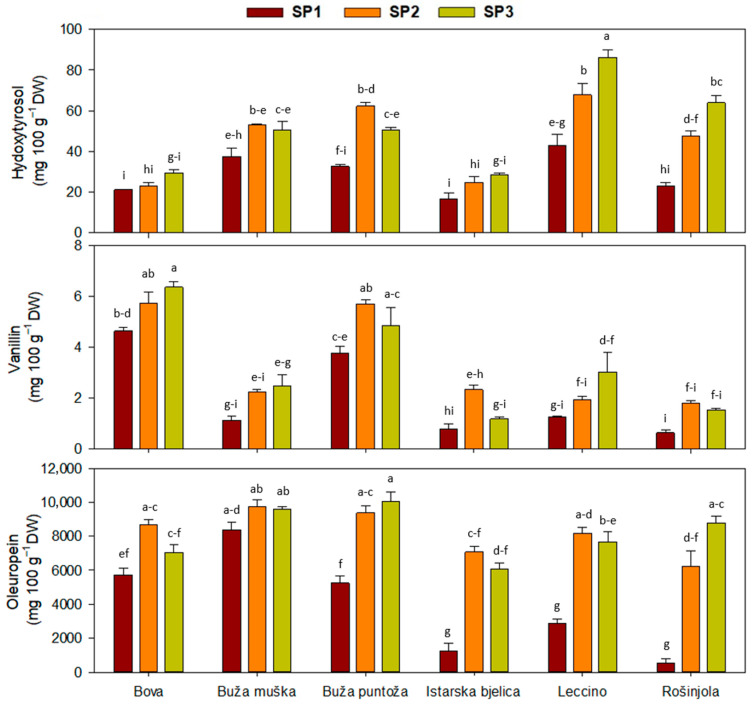
Concentration of simple phenols and secoiridoids in olive leaves, when interaction between cultivars and sampling periods (SP) was significant in two-way ANOVA (Table 1). Different letters represent statistically significant differences between means ± standard errors (n = 3) as tested by Tukey’s post-hoc test at *p* ≤ 0.05. Sampling periods were: harvesting in October (SP1), dormancy in January (SP2), and pruning in March (SP3). The six olive cultivars were: Bova, Buža muška, Buža puntoža, Istarska bjelica, Leccino, and Rošinjola.

**Figure 2:**
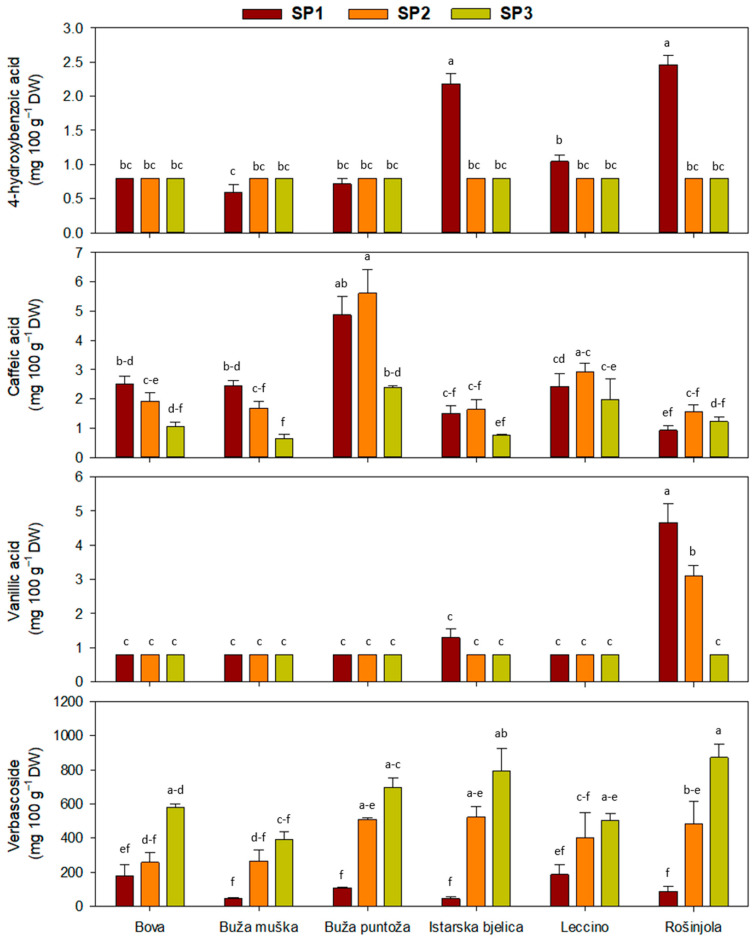
Concentration of phenolic acids in olive leaves, when interaction between cultivars and sampling periods (SP) was significant in two-way ANOVA (Table 2). Different letters represent statistically significant differences between means ± standard errors (n = 3) as tested by Tukey’s post-hoc test at *p* ≤ 0.05. Sampling periods were: harvesting in October (SP1), dormancy in January (SP2), and pruning in March (SP3). The six olive cultivars were: Bova, Buža muška, Buža puntoža, Istarska bjelica, Leccino, and Rošinjola.

**Figure 3:**
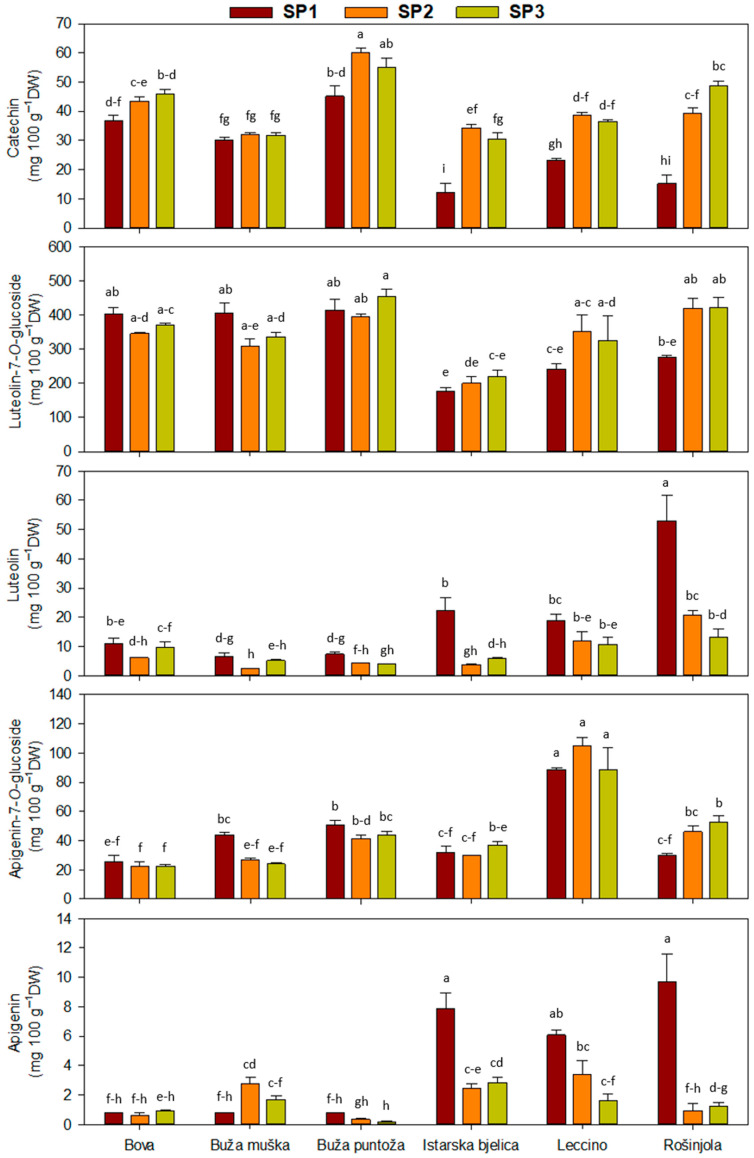
Concentration of phenolic acids in olive leaves, when interaction between cultivars and sampling periods (SP) was significant in two-way ANOVA (Table 3). Different letters represent statistically significant differences between means ± standard errors (n = 3) as tested by Tukey’s post-hoc test at *p* ≤ 0.05. Sampling periods were: harvesting in October (SP1), dormancy in January (SP2), and pruning in March (SP3). The six olive cultivars were: Bova, Buža muška, Buža puntoža, Istarska bjelica, Leccino, and Rošinjola.

The concentrations of simple phenols and secoiridoids, phenolic acids, and flavonoids are presented in Table 1, Table 2 and Table 3, while multiple comparisons of significant interactions of cultivar and sampling period, as the main factors, are presented in Figure 1, Figure 2 and Figure 3 (see Supplementary Table S2 for full ANOVA results, including F values, degrees of freedom, and *p* values). As shown in Table 1, only for tyrosol and total phenol levels, there was no interaction effect.

The lowest leaf total phenol levels were detected in SP1, while insignificant differences among the other two sampling periods (SP2 and SP3) were found. Although the analysis of variance showed a significant effect of sampling period on tyrosol concentration, the post hoc test did not show differences among sampling periods for this phenol (significance for sampling period in analysis of variance was *p* = 0.45); significant differences were recorded among the cultivars ‘Bova’ (5.3 mg 100 g^−1^ DW), ‘Buža muška’ (8.2 mg 100 g^−1^ DW), and ‘Leccino’ (12.2 mg 100 g^−1^ DW). Cultivar ‘Leccino’ exhibited significantly greater values compared to the other cultivars. Two-way interaction for hydroxytyrosol demonstrated that leaves of some cultivars had recorded higher values in SP3 than SP1 (Figure 1). This trend was most pronounced for the cultivars ‘Leccino’ and ‘Rošinjola’. The highest hydroxytyrosol levels were recorded in the ‘Leccino’ cultivar at SP3 (86.3 mg 100 g^−1^ DW), while the lowest ones were determined in the ‘Istarska bjelica’ cultivar at SP1 (16.7 mg 100 g^−1^ DW). ‘Bova’ and ‘Istarska bjelica’ exhibited lower values at SPs 2 and 3 compared to the other cultivars (Figure 1).

Similarly to hydroxytyrosol, the vanillin content was influenced by the two-way interaction of the main factors, cultivar and sampling period (Figure 1). As shown in Figure 1, two cultivars, ‘Buža puntoža’ and ‘Bova’, consistently exhibited notably higher values in all SPs. The vanillin content at SP3 was higher compared to SP1, while in the ‘Rošinjola’ cultivar, SP1 displayed a significantly lower vanillin content compared to the other two SPs. Analysis of variance for oleuropein revealed that there were significant differences in the oleuropein content between SPs, with the lowest content determined at SP1 (Table 1). Two-way interaction largely confirmed this trend, except for cultivars ‘Buža muška’ and ‘Bova’ (Figure 1). Particularly noteworthy were the low SP1 values recorded for cultivars ‘Istarska bjelica’, ‘Leccino’, and ‘Rošinjola’, which were significantly lower compared to the oleuropein contents for cultivars ‘Buža muška’, ‘Buža puntoža’, and ‘Bova’.

A significant two-way interaction of SP and cultivar was observed for all phenolic acids, with the exception of ferulic acid, whose content was not affected by either factor (Table 2).

The highest caffeic acid content was determined in ‘Buža puntoža’ at SP 2 (Figure 2). For verbascoside, a general increasing trend was observed from SP1 to SP3 (Figure 2). Significant differences between SP1 and SP2 were detected in cultivars ‘Buža puntoža’, ‘Bova’, ‘Istarska bjelica’, and ‘Rošinjola’. Vanillic and 4-hydroxybenzoic acids exhibited similar response patterns, as indicated by the significant two-way interaction of the main factors (SP and cultivar). A decline in the vanillic acid content from SP1 to SP3 was observed for ‘Rošinjola’ (Figure 2). There was no difference between different cultivars in the same SP or within the same cultivar in different SPs regarding the ferulic acid concentration. Furthermore, significant differences in Cv. × SP interaction were only recorded between Buža puntoža × SP2 and Rošinjola × SP1 (Table 2).

The flavonoid content was, in most cases, significantly affected by a two-way interaction (Table 3). Differences in the rutin content among SP1 and 3 were recorded (Table 3). ‘Leccino’ had substantially higher values of this flavonoid compared to Buža muška, Buža puntoža, and ‘Rošinjola’. For the cultivar ‘Buža puntoža’, as the cultivar with the highest catechin concentration, only a significant difference was found between SP1 and SP2. Overall, the highest catechin concentration was recorded at SP2 for the ‘Buža puntoža’ cultivar (60.20 mg 100 g^−1^ DW). (Figure 3).

The luteolin content differed significantly between cultivars, among SP1 and 3 only for ‘I. bjelica’ and ‘Rošinjola’. The highest luteolin content was recorded for ‘Rošinjola’, at SP1 (Figure 3). ‘Leccino’ exhibited significantly higher apigenin-7-*O*-glucoside contents (in SP1, 2, and 3), compared to all the other cultivars. Similar to luteolin, the highest apigenin levels were recorded in cultivars ‘Istarska bjelica’ and ‘Rošinjola’, at SP1 (Figure 3). Generally, it can be concluded that the apigenin content decreased from SP1 to SP3 in half of the investigated cultivars (Figure 3).

### 2.2. Olive Leaf Nutrient Concentrations

**Figure 4:**
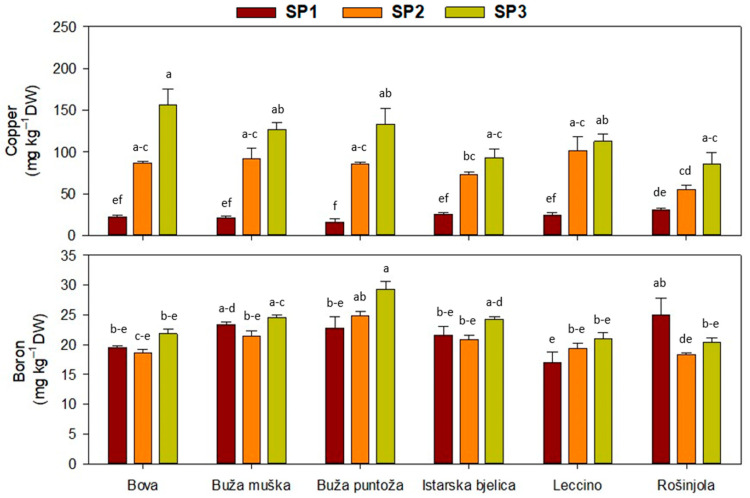
Concentration of micronutrients in olive leaves, when interaction between cultivars and sampling periods (SP) was significant in two-way ANOVA (Table 3). Different letters represent statistically significant differences between means ± standard errors (n = 3) as tested by Tukey’s post-hoc test at *p* ≤ 0.05. Sampling periods were: harvesting in October (SP1), dormancy in January (SP2), and pruning in March (SP3). The six olive cultivars were: Bova, Buža muška, Buža puntoža, Istarska bjelica, Leccino, and Rošinjola.

Olive leaf nutrient analysis revealed that both SP and cultivar had a significant impact on mineral composition. Results of the analysis of variance are summarised in Table 4 (see Supplementary Table S2 for full ANOVA results, including F values, degrees of freedom, and *p* values). Interactions of the main factors were not significant for most nutrients; with regard to SP as a factor, a decreasing trend from SP1 to SP3 was observed for P, K, and Fe concentrations (Table 4). ‘Buža puntoža’ exhibited the highest P levels, compared to all the other genotypes (Table 4). Finally, a significant interaction effect was observed for Cu, showing an increase in leaf Cu concentration from SP1 to SP3 for all the studied cultivars (Figure 4).

### 2.3. Separation of Cultivars and Sampling Periods Based on Linear Discriminant Analysis (LDA)

**Figure 5:**
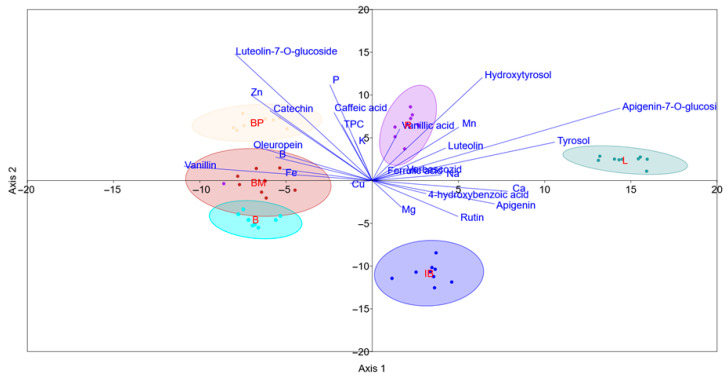
Separation of olive cultivars ‘Bova’, ‘Buža muška’, ‘Buža puntoža’, ‘Istarska bjelica’, ‘Leccino’, and ‘Rošinjola’ based on linear discriminant analysis of a dataset comprising phenol profile and element concentrations in leaves. TPC—total phenol concentration.

The results demonstrated a clear separation of cultivars, particularly ‘Leccino’, ‘Rošinjola’, and ‘Istarska bjelica’ (Figure 5). The first two axes accounted for 72.3% of the variation. The confusion matrix indicated 100% of correctly classified datapoints, following the jackknife cross-validation procedure. LDA analysis revealed that based on the provided data, the cultivars ‘Buža muška’, ‘Bova’, and ‘Buža puntoža’ were relatively similar in terms of phenolic and mineral concentrations. Cultivar ‘Leccino’ was the most divergent, compared to the other three (Figure 5).

**Figure 6:**
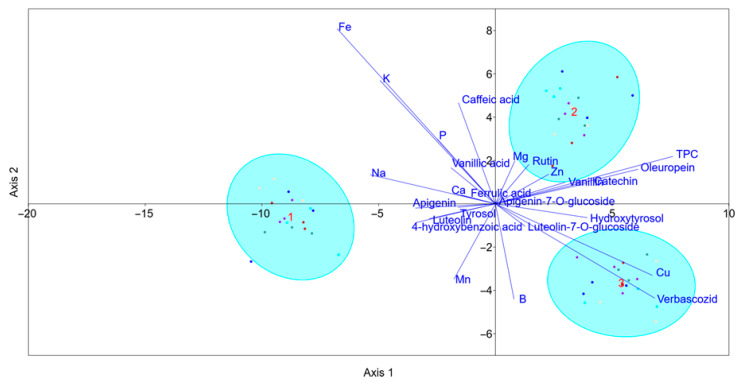
Separation of sampling periods based on linear discriminant analysis and a dataset containing phenolic compounds and element concentrations in olive leaves. TPC—total phenol concentration. Sampling periods were harvesting in October—SP1 (1), dormancy in January—SP2 (2), and pruning in March—SP3 (3).

A scatterplot of the grouping based on SP performed by LDA is presented in Figure 6. Separation without overlap has been achieved among all three SPs with 100% of explained variance. Axis 1 accounted for 76.1% of the variance, while Axis 2 accounted for 23.9%.

The confusion matrix obtained using the jackknife cross-validation procedure indicated that 100% of the datapoints were correctly classified. According to linear discriminant coefficients for Axis 1, the most important variables are TPC (total phenol concentration), oleuropein, verbascoside, luteolin-7-*O*-glucoside, apigenin-7-*O*-glucoside, and rutin. The most important variables based on Axis 2 were TPC, oleuropein, verbascoside, luteolin-7-*O*-glucoside, and Cu. These results suggest that the separation of SP1 and 3 was mostly based on Axis 1, while the separation of SP1 and 3 from SP2 was based on Axis 2. Iron, K, and P levels contributed to the separation of SP1 and 3, with higher concentrations at SP1 compared to SP3. Similarly, verbascoside and Cu, which were negatively correlated with the mentioned nutrients, also showed a similar trend.

### 2.4. Analysis of Potential Links Between Phenolic Compounds and Nutrients Using Pearson’s Correlation Matrix

The correlation matrix between phenolic and nutrient concentrations is shown in Table 5. Some of the strongest correlations were observed between: (i) P and caffeic acid (0.83, *p* ≤ 0.01), (ii) Cu and TPC (0.79, *p* ≤ 0.001), (iii) Na and verbascoside (0.72, *p* ≤ 0.01) and TPC (0.72, *p* ≤ 0.01), and (iv) K and verbascoside (0.75, *p* ≤ 0.001).

Among nutrients, the highest number of correlations was found for Cu. The oleuropein concentration correlated positively with Cu (Table 5). Verbascoside was negatively correlated with K, while its correlation with Cu was positive. The additional correlation analysis for each cultivar separately revealed negative correlations between oleuropein and P or K, verbascoside and P or K, and TPC and P or K, only for ‘Rošinjola’. There were positive correlations between verbascoside and B for ‘Leccino’, ‘Buža puntoža’, and ‘Bova’ (Table S1).

## 3. Discussion

Profiles of olive leaf phenols are generally affected by a large number of different factors, such as geographical origin, moisture concentration, cultivar, tree and leaf age, or fertilisation practices [4,20,21,22,23,24,25,26,27]. Recent studies also emphasise that ecological and phenological factors greatly affect the phenolic compounds in different crops. This interaction is essential for determining the ideal harvest time to maximise secondary metabolite content and the therapeutic properties of the plant material, as well as plant resilience to different abiotic and biotic stressors [4,6,7,28]. Likewise, nutrient uptake has been previously shown to vary among Portuguese olive cultivars [29]. In contrast, the effect of genotype and season on nutrient uptake by some Greek olive cultivars has been described by Chatzistathis et al. [30], Chatzistathis et al. [31], and Manolikaki et al. [32]. Although numerous authors have investigated seasonal variations in antioxidant compounds in olive leaves, they did not assess the nutrient status [33,34]. Therefore, while their findings confirm the existence of seasonal trends in phenolic content, the potential influence of nutrient dynamics remains to be elucidated.

Olive leaf phosphorus (P), potassium (K), copper (Cu), and boron (B) concentrations were previously correlated with the levels of important phenolic compounds, particularly oleuropein and verbascoside [22]. We can assume that the seasonal variations in phenolic composition could be attributed to the dynamics of some nutrients, such as potassium (K) and manganese (Mn). Both nutrients are known to affect photosynthetic activity and the biosynthesis of phenolic compounds [35]. Apart from the environmental effects, the efficiency of K and Mn uptake and translocation may differ among cultivars, influenced by variations in root system architecture, nutrient use efficiency, and other physiological functions [30,36,37]. However, although we have previously noted Mn’s impact on olive leaf secondary metabolism, particularly related to oleuropein and luteolin-7-*O*-glucoside modulations [38], in this research, only a strong Mn–tyrosol correlation has been recorded.

Based on the interpretations of nutrient concentrations in olive leaves provided by Fernández-Escobar [39], nutrient levels in our study were all within the adequate range, except for K, which was below, for all cultivars. Deficiencies in K are frequently found in orchards situated on calcareous soils and under rainfed conditions [39] and are not easily corrected because K fertilisers are taken up in small amounts by deficient or water-stressed trees [40]. It has been stated previously that the main nutritional imbalance that could affect the majority of olive orchards is potassium deficiency [39]. The constant decrease in K concentration across sampling periods is consistent with the fact that winter temperatures lower K mobility and availability [41]. Such a pattern of K concentration decreasing from October to March has been previously reported by Lukić et al. [42]. The significant negative correlation observed between K concentration and verbascoside content across all cultivars and sampling periods in our data likely indicates a stress-induced reaction to K deficiency. This finding corresponds with the established behaviour of phenylalanine ammonia-lyase (PAL), a crucial enzyme in the phenylpropanoid pathway, which is triggered by both nitrogen and potassium deficiencies [43]. The highest K content was observed in ‘Buža puntoža’ and ‘Buža muška’. Importantly, significant negative correlations between K and oleuropein, verbascoside, as well as TPC, were exclusively identified for ‘Rošinjola’, which recorded some of the lowest K concentrations. For all of the cultivars, including the previously mentioned one, such a negative correlation was recorded on much younger olive trees, only for K and verbascoside [22].

Decreases in P concentration from SP1 to SP3 (from October to March) have been reported previously [41]. This decrease in P concentration in March and April coincides with the development of flower buds and inflorescences; higher P concentrations during the winter and lower in March have also been reported by Fahmy and Nasrallah, along with K and Fe, which is also in concordance with our results [44]. While earlier studies on younger trees grown nearby revealed a strong negative correlation between P and phenolic variables, such as oleuropein, verbascoside, and TPC [22], our research only identified these correlations in certain cultivars (‘Rošinjola’ and ‘Buža puntoža’), whereas others (‘Leccino’, ‘Istarska bjelica’, ‘Buža muška‘, and ‘Bova’) showed different patterns (Table S1). Notably, a robust positive correlation with caffeic acid was found for all cultivars, suggesting that interactions between nutrients and phenols are not only specific to each cultivar but also to individual compounds and can be influenced by different factors such as plant age [5]. Moreover, caffeic acid appears to play a crucial role in how plants respond to abiotic stress, contributing to various functions, such as regulating biosynthesis pathways, boosting antioxidant enzyme expression, and triggering the phenylpropanoid pathway [45]. In general, it is well known that P is essential for ATP, driving phosphorylation within the shikimate pathway. This process begins when phosphoenolpyruvate (PEP), an intermediate from glycolysis, reacts with erythrose-4-phosphate (E4P), leading to the synthesis of aromatic amino acids, such as phenylalanine or tyrosol, which serve as a precursor for phenolic compound biosynthesis [46].

Higher concentrations of Cu in March, as well as higher concentrations of K in October, could be related to standard fertilisation (K) and plant protection (Cu) practices [22]. It can be noted that recorded concentrations are over the limit for excess Cu (>78 mg/kg) stated by Sotiropoulos et al. [47] and higher compared to our previous research for the pruning-related period [22,48] but without visible signs of phytotoxicity. Strong positive correlations between Cu and oleuropein (0.65, *p* = 0.004), as well as with verbascoside (0.70, *p* = 0.001) and TPC (0.79, *p* < 0.001) have been previously observed in olive leaves [22,42]. Different classes of Cu metalloenzymes, such as PPO (polyphenol oxidases), SOD (superoxide-dismutase), or laccase, are directly or indirectly involved in plant phenolic metabolism [49,50].

Despite varying conclusions from previous studies concerning olive leaf B concentration and its correlations with olive leaf phenolic compounds [22,51], in our research, a significant positive correlation with verbacoside concentration was noted only for ‘Leccino’, ‘Buža puntoža’, and ‘Bova’. Cultivar, which is closely related to plant nutrient uptake and translocation, is one of the main factors affecting phenolic variability in olive leaves [52,53]. Still, environmental conditions along with phenological phase, which are the core of sampling period definition, also significantly impact this variability. In this research, all nutrient concentrations in olive leaves, with the exception of Cu and B, were influenced only by these two main factors. Paskovic et al. [27] reported almost the same result in their research on ‘Leccino’, ‘Drobnica’, ‘Istarska bjelica’, ‘Lastovka’, and ‘Oblica’ cultivars.

Previous studies have generally reported higher oleuropein concentrations during colder periods and/or the pruning season [4,54,55]. In contrast, Ugolini et al. [56] reported that oleuropein accumulation increased under warmer conditions, while Dias et al. [57] found a similar response under UV-B stress in growth chamber experiments. In our study, February temperatures were 1.3 °C below the 30-year average, with negative temperatures in the weeks before sampling [22,58], which likely contributed to the highest oleuropein levels recorded in March (SP3). This sampling is in line with the phenological phase of swollen buds, which corresponds to stage 51 of the BBCH scale, marking the transition from eco-dormancy to active growth defined as ‘spring flush’, when buds begin to swell and metabolic reprogramming occurs [59]. The occurrence of peak oleuropein levels aligning with this phenological stage may suggest that the developmental status of buds plays an important role in phenolic accumulation. Supporting this, Noronha et al. [60] documented substantial metabolic reprogramming which includes sugar mobilisation, cell wall changes, and cell division at bud burst in grapevine woody tissues, highlighting the broad biological relevance of this transition stage.

Overall, Lorini et al. [61] demonstrated that summer conditions in olive leaves favour the accumulation of other phenolics, such as apigenin, hydroxybenzoic acid, luteolin, and tyrosol. Moreover, although a higher phenolic content is generally associated with lower rainfall levels [62], Alderotti et al. [63] reported cultivar-specific responses to water deficit, with variable effects on oleuropein and flavonoids.

In our research, across all the cultivars and sampling periods, oleuropein emerged as the most prevalent phenolic compound in olive leaves, underscoring its prominent role in the olive leaf phenolic profile, as widely documented in existing literature [27,64,65,66,67]. Among flavonoids, luteolin-7-*O*-glucoside was the most abundant, consistent with earlier research [53,68], but there was no seasonal variation within each of the selected cultivars. On the contrary, the same luteolin glucoside levels tend to be highest in the spring period due to the latest findings for Italian cultivars [56]. However, it seems that the complex interplay between heat stress and UV-B radiation has a significant role in previously mentioned luteolin glucoside concentration in olive leaves [57]. Interestingly, Mir-Cerda et al. [69] stated that the phenolic composition of olive leaves is mainly influenced by location and season, with autumn being the optimal period for harvesting due to higher phenolic content, notably luteolin-7-*O*-glucoside and oleuropein, while a small sample size and overlapping effects of origin and season limited the study of cultivars. Although present, drastic differences between cultivars in the verbascoside concentration were not observed, as reported by Orak et al. [70], and were mainly related to the lower concentration in the pruning period between ‘Buža muška’ and almost all cultivars except for ‘Leccino’. Still, verbascoside ranked as the most prevalent phenolic acid, corroborating other published findings [42].

Numerous studies have reported seasonal variations in different phenolic compound concentrations in olive leaves [22,42]. Lukić et al. [42] noted that the concentrations of hydroxytyrosol, vanillin, oleuropein, total phenols, verbascoside, and catechin were higher during the pruning period, compared to harvest. Similar seasonal trends for hydroxytyrosol, oleuropein, verbascoside, and catechin were validated by Polić Pasković et al. [22] and Pasković et al. [27]. These insights indicate the need for a more thorough analysis of the relationship between cultivar and sampling period, especially regarding these compounds.

Diab et al. [33] examined seasonal fluctuations in oleuropein levels in olive leaves across various cultivars (‘Kalamata’, ‘Picual’, ‘Koroneiki’, ‘Agizy’, and ‘Manzanillo’). They found that approximately half of them exhibited reduced oleuropein concentrations in spring, while ‘Picual’, and ‘Manzanillo’, had elevated levels in winter. Most cultivars in this study showed patterns similar to ‘Picual’, and ‘Manzanillo’. However, the highest oleuropein levels, regardless of the sampling period, were observed in ‘Buža muška’. This consistency in oleuropein concentrations aligns well with previous findings in the ‘Istarska bjelica’, cultivar, indicating a unique metabolic reaction to environmental conditions [27]. In this research, ‘Istarska bjelica’, along with ‘Buža puntoža’, ‘Leccino’, and ‘Rošinjola’, exhibited temporal variations in secoiridoid content, which matched results from parallel studies on younger trees of the same cultivars. Significantly, substantial differences were noted during the pruning period, particularly concerning oleuropein concentrations in leaves [22].

Among all the examined cultivars, only ‘Rošinjola’, exhibited complete consistency in the differences noted between the harvest and pruning periods for the majority of phenolic compounds, with markedly higher values for oleuropein, verbascoside, hydroxytyrosol, and catechin during the pruning period, as well as vanillic acid, 4-hydroxybenzoic acid, luteolin, apigenin, and TPC concentrations that peaked during the harvest period [22]. Research on two *Prunus avium* L. cultivars revealed that environmental drivers such as temperature, sunshine duration, and rainfall have a significant impact on several groups of phenolic compounds in sweet cherry leaves. Among them, flavonoid concentrations have been shown to increase at the beginning and end of the season [6]. During the early vegetative stage, particularly in shaded, moist habitats, the accumulation of beta-hydroxycinnamic acids reached the highest levels in *Epilobium hirsutum* L. leaves, a perennial medicinal plant similar to olive leaves, known for its considerable pharmaceutical value. Furthermore, its higher flavonoid content was recorded alongside plant maturation. Thus, the highest rates of flavonoid glycosides occurred during the hairy willow herb flowering period (June-August) [71]. Interestingly, although the ‘Rošinjola’ cultivar is regarded as *Spilocea oleaginea*-tolerant, only oleuropein levels in SP3 set it apart from susceptible cultivars like ‘Istarska bjelica’ [72], within a specific list of phenolic compounds previously associated with olive leaf spot cultivar’s resistance by other authors [15]. This underscores a critical need for further research into the phenolic leaf profiles of various Croatian olive cultivars and their resistance to common olive pathogens.

Luteolin-7-*O*-glucoside, recognised as a flavonoid with the highest concentration in this research, consistently showed lower levels in ‘Istarska bjelica’, compared to ‘Buža puntoža’, across all the sampling periods. This finding aligns with earlier reports of cultivar-specific differences [22]. Similarly, apigenin-7-*O*-glucoside levels were consistently highest in ‘Leccino’, regardless of the sampling period, which is consistent with previous studies [22,27].

A likely reason for the notable cultivar-specific variation in phenolic compound concentrations is the differing activity of two key enzymes involved in PAL, which facilitates phenol biosynthesis, and PPO, which participates in phenol degradation. Previous research has shown that cultivars with elevated total phenolic concentrations typically exhibit increased PAL activity and decreased PPO activity. In contrast, the opposite trend is observed in cultivars with lower phenolic levels [54]. These enzymatic patterns may also correlate with seasonal variations, since total phenolic concentrations were found to be lower in October (SP1) than in March (SP3), indicating reduced PAL activity and/or increased PPO activity in the earlier sampling period, which is in accordance with the data published by Ortega-García and Peragón [54].

Based on almost the same set of variables and using multivariate techniques such as partial least squares–discriminant analysis (PLS-DA) and forward stepwise discriminant analysis (SLDA), discrimination is possible between the sampling times of October and March and October-January-March [22,42]. Similar to this research, we were able to discriminate between three sampling times with no overlap between them. Discrimination between genotypes, based on multivariate analysis, was also performed, and separation of cultivars was observed without overlap (except for slight overlap of ‘Buža muška’ and ‘Bova’, cultivars showing their similarity). This was in accordance with the study of Lukić et al. [42], who used SLDA and discriminated between genotypes, based on phenols and minerals, with only slight overlap between some cultivars. In addition, although ‘Buža muška’, was not strictly mentioned, it has been pointed out that some other genotypes, such as ‘Buža’, ‘Buža ženska’, and ‘Buža puntoža’, share the same word in their name, which is “Buža” and have been identified as homonyms (same name for different cultivars) [73]. Moreover, Ercisli et al. [74] concluded that ‘Buža muška’ and ‘Levantinka’ are the most closely related cultivars as they produced identical SSR profiles. Podgornik et al. [75] stated that samples from the Croatian region of Istria, identified as ‘Buža muška’, ‘Porečka Rosulja’, and ‘Črna’, share an identical genetic profile, while significant differences in allele length were observed in the other “Buža” group of trees, such as ‘Buža ženska’ and ‘Buža puntoža’. Therefore, a separation between ‘Buža muška’ and ‘Buža puntoža’ was expected. This, in summary, highlights the importance of multivariate techniques towards discriminating olive cultivars grown in the same orchard, under the same environmental conditions, which are modulated by different sampling periods. In practice, this approach could be used to identify the appropriate cultivar for harvesting leaves rich in phenols, or a group of phenols, as well as support future smart use of olive by-products.

## 4. Materials and Methods

### 4.1. Olive Leaf Sampling

The leaves were collected from mature, 30-year-old olive trees grown as part of the experimental collection of the Institute of Agriculture and Tourism, Poreč, Croatia (45°22′ N; 13°60′ E). The rain-fed olive grove is situated in Rhodic cambisol soil, with slightly basic pH (7.8 in H_2_O and 7.2 in KCl), 3.8% organic matter, 0.2% nitrogen (N) concentration, and 80 mg kg^−1^ and 338 mg kg^−1^ plant available phosphorus (P) and potassium (K), respectively, analysed as it was previously described [20]. Standard fertilisation and integrated pest management practices were followed annually [22,76]. The climate in this area is classified as Cfa according to the Köppen climate classification, and temperatures and rainfall were in accordance with a published study on 10-year-old olive trees grown in the area [22].

Only three healthy, well-developed trees of six cultivars, namely ‘Bova’, ‘Buža muška’, ‘Buža puntoža’, ‘Rošinjola’, ‘Istarska Bjelica’, and ‘Leccino’, were selected for leaf sampling. One hundred leaves were randomly sampled from the middle canopy section of each tree, ensuring equal distribution around the canopy during each sampling period: during the harvest period in October 2017 (SP1), during the dormancy period in January 2018 (SP2), and during pruning in March 2018 (SP3). The experiment was set in a completely randomised design, with each of the six cultivars represented by three trees, totalling 18 trees in the study.

After each sampling, leaves were carefully washed in the laboratory, first with tap water, followed by 1% acetic acid solution in deionised water, and finally with deionised water. Leaves were then air-dried to a constant mass and ground into a fine powder using a Retsch ZM 200 mill (Retsch GmbH, Haan, Germany) before analysis.

### 4.2. Chemicals and Standards

Methanol (MeOH) and acetonitrile (AcN) were obtained from Merck (Darmstadt, Germany), while phosphoric acid was acquired from Sigma-Aldrich (St. Louis, MO, USA). Standards for apigenin, apigenin-7-*O*-glucoside, catechin, hydroxytyrosol, luteolin, luteolin-7-O-glucoside, oleuropein, rutin, tyrosol, verbascoside, vanillin, 4-hydroxybenzoic, caffeic, ferulic, and vanillic acids were purchased from Extrasynthese (Genay, France). Deionised water was produced using a Siemens UltraClear system (Siemens AG, München, Germany). A multi-element standard solution from Perkin Elmer (NexION Setup Solution, Waltham, MA, USA) was used. Argon, with a purity of 6.0, was supplied by Messer (Bad Soden, Germany) for forming plasma in the inductively coupled plasma mass spectrometer (ICP-MS) analysis, while acetylene was provided by Messer Croatia Plin d.o.o. (Zaprešić, Croatia).

### 4.3. High-Performance Liquid Chromatography (HPLC) and Elemental Analysis

Dried and ground leaf powder was extracted with methanol:water (80:20, *v*:*v*) solution (HPLC grade; Merck, Darmstadt, Germany) by ultrasonic treatment (20 min) in an ultrasonic bath (Sonorex Digitec; Bandelin electronic, Berlin, Germany). The extracts were centrifuged at 5000 rpm for 5 min (Domel Centric 350; Železniki, Slovenia) and filtered using a cellulose acetate syringe filter with 0.45 μm pores prior to HPLC analysis. The method was adapted from our previous work [22,23] to maintain consistency with olive leaf phenolic profiling studies. It should be noted that the extraction procedure used in this study was not specifically optimised to maximise the absolute yield of phenolic compounds. Rather, a conventional methanol:water solvent system was selected to ensure methodological consistency and direct comparability with previous agronomic studies on olive leaf phenolics. Nevertheless, the growing body of literature on green extraction techniques, particularly NADES-based approaches [77,78], suggests that future investigations could substantially increase phenolic recovery while reducing environmental impact. Phenolic compounds were determined by the HPLC Ultimate 3000 System with a UV/VIS detector capable of simultaneous measurement at 4 different wavelengths (ThermoFisher Scientific, Waltham, MA, USA). Phenols were separated using a Lichrospher 100 RP-18 (250 mm × 4 mm, 5 µm) analytical column with a pre-column Lichrospher 100 (4 mm × 4 mm, 5 µm), both supplied by Agilent Technologies (Santa Clara, CA, USA). For the chromatographic separation, 0.2% phosphoric acid (Sigma-Aldrich, St. Louis, MO, USA) in water was used as solvent A; and MeOH: ACN (1:1, *v*:*v*; Merck, Darmstadt, Germany) as solvent B. The solvent gradient was as follows: 10% B 0–0.5 min; 10–16.5% B 0.5–25 min; 16.5–30% B 25–80 min; 30–100% B 80–95 min; 100% B 95–100 min; 100–10% B 100–102 min; 10% B 102–105 min; followed by an equilibration time of 10 min. A volume of 10 µL of each extract was injected into the system and eluted with a flow rate of 0.8 mL/min at 25 °C. UV/Vis detection was optimised for best sensitivity for each compound, so the phenols were grouped at the available 4 wavelengths as follows: 4-hydroxybenzoic acid, luteolin-7-*O*-glucoside, oleuropein, and vanillic acid (measured at 250 nm); apigenin-7-*O*-glucoside, catechin, hydroxytyrosol, tyrosol, and vanillin (measured at 280 nm); apigenin, caffeic acid, ferulic acid, and verbascoside (measured at 305 nm); luteolin and rutin (measured at 370 nm). All phenolic standards were purchased from Extrasynthese (Genay, France). Phenols were identified by comparison of their retention times with those of standards and quantified using the external standard method. The calibration curves used for concentration calculation (R^2^ ≥ 0.999) consisted of five calibration levels made by appropriate dilutions of the stock standard solutions. Additionally, elemental analysis of dried olive leaves was performed using Inductively Coupled Plasma Mass Spectrometry (ICP-MS) and atomic absorption spectrometry (AAS) as described in detail previously [22,23].

### 4.4. Statistical Analysis

A two-way analysis of variance (two-way ANOVA) was conducted to examine the effects of the main factors, cultivar, and sampling period, along with their interaction.

The assumptions of normality of residuals and homogeneity of variances were evaluated for each variable using the Shapiro–Wilk test and Levene’s test (Brown–Forsythe version).

Data that violated the normality assumption (*p* < 0.05) were transformed (logarithmic or inverse square root transformation) before analysis. Homogeneity of variances was satisfied for all variables in the original dataset. Variables that failed to meet the assumption of normality even after transformation (4-hydroxybenzoic acid, vanillic acid, vanillin, ferulic acid, rutin, luteolin-7-O-glucoside, and magnesium) were interpreted with caution.

For consistency and comparability, all variables (transformed or untransformed) were analysed using the same statistical method, noting that ANOVA is generally robust to moderate deviations from normality, especially in balanced designs (n = 3 per group) [79]. ANOVA and Tukey’s post-hoc test were conducted on transformed data when necessary, with results presented as means ± SE of untransformed values using the TIBCO Statistica 14.1.0.8 software (TIBCO StatSoft^®^, Palo Alto, CA, USA).

Shapiro–Wilk and Levene’s median tests for each variable were conducted in R, version 4.4.1 [80], using RStudio [81]. The car package [82] was used for Levene’s test, while the base functions aov and shapiro.test were applied for ANOVA and Shapiro–Wilk testing. Linear discriminant analysis (LDA) was conducted on the scaled data using PAST software v4.16c, as well as for Pearson’s correlation analysis [83].

## 5. Conclusions and Future Directions

Copper exhibited strong positive correlations with oleuropein and verbascoside, the most abundant phenolic compounds in olive leaves, as well as total phenolic leaf concentrations, irrespective of cultivar. Boron, on the other hand, showed significant correlations with verbascoside only in the cultivars ‘Leccino’, ‘Buža puntoža’, and ‘Bova’.

The study confirmed significant seasonal variation in olive leaf phenolic concentrations across the three sampling periods, which aligns with recent findings that environmental factors and phenological stages strongly influence the concentrations of phenolic compounds in leaves of different horticultural plants. In general, the total phenolic content was higher during the cold winter months and lower in the warmer harvest period, regardless of the selected cultivars. The oleuropein concentration was significantly affected by cultivar and sampling time interactions, revealing the importance of genotype in modulating seasonal phenolic responses. Cultivar ‘Buža muška’ consistently exhibited higher concentrations throughout all the sampling periods, indicating it as a promising candidate for enhancing leaf oleuropein levels. Notably, ‘Rošinjola’ displayed a distinctive seasonal phenolic pattern that significantly differs from harvest to the pruning period and aligns well with our previous research on the temporal dynamics of phenolic compounds in this cultivar.

The results of this study reveal considerable variability in phenolic composition among Croatian olive cultivars, supporting the need to further investigate the link between genotypic resistance, olive leaf fungal communities, phenolic profiles, and disease incidence under field conditions.

## Figures and Tables

**Table 1:** Concentration (mg 100 g^−1^ dry matter) of simple phenols, secoiridoid oleuropein, and total phenols in leaves of six olive cultivars (Cv.) collected at three different sampling periods (SPs), harvesting in October (SP1), dormancy in January (SP2), and pruning in March (SP3), and two-way ANOVA results.

Main Effect	Simple Phenols			Secoiridoids	
	Hydroxytyrosol	Tyrosol	Vanillin	Oleuropein	Total Phenol Concentration
**Cultivar (Cv.)**					
‘Bova’	25 ± 2 ^c^	5.3 ± 0.8 ^c^	5.6 ± 0.3 ^a^	7162 ± 463 ^bc^	4247 ± 158 ^ab^
‘Buža muška’	47 ± 3 ^b^	8.2 ± 0.5 ^b^	1.9 ± 0.3 ^b^	9218 ± 282 ^a^	4460 ± 304 ^a^
‘Buža puntoža’	484 ± 4 ^b^	6.7 ± 0.5 ^bc^	4.8 ± 0.4 ^a^	8218 ± 784 ^ab^	4630 ± 380 ^a^
‘Istarska bjelica’	23 ± 2 ^c^	6.7 ± 0.6 ^bc^	1.4 ± 0.2 ^b^	4801 ± 923 ^e^	3878 ± 297 ^b^
‘Leccino’	66 ± 7 ^a^	12.2 ± 0.5 ^a^	2.1 ± 0.3 ^b^	6239 ± 869 ^cd^	4284 ± 368 ^ab^
‘Rošinjola’	45 ± 6 ^b^	7.1 ± 0.3 ^bc^	1.3 ± 0.2 ^b^	5179 ± 1250 ^de^	4626 ± 315 ^a^
**Sampling period (SP)**					
SP1	28.9 ± 10.8 ^c^	8.5 ± 0.6	2.0 ± 0.4 ^b^	4005 ± 673 ^b^	3230 ± 103 ^b^
SP2	46.4 ± 18.0 ^b^	7.4 ± 0.8	3.3 ± 0.4 ^a^	8209 ± 345 ^a^	4933 ± 111 ^a^
SP3	51.4 ± 20.9 ^a^	7.2 ± 0.6	3.2 ± 0.5 ^a^	8194 ± 369 ^a^	4899 ± 123 ^a^
**Cv.**	***	***	***	***	**
**SP**	***	*	***	***	***
**Cv. × SP**	***	ns	**	***	ns

Shown are means ± standard errors (n = 3). Different letters in a column represent statistically significant differences between mean values for each main effect at *p* ≤ 0.05 obtained by a two-way ANOVA and Tukey’s post hoc test (ns, not significant; *, *p* ≤ 0.05; **, *p* ≤ 0.01; ***, *p* ≤ 0.001).

**Table 2:** Concentration (mg 100 g^−1^ dry matter) of phenolic acids in leaves of six olive cultivars (Cv.) collected at three different sampling periods (SPs), harvesting in October (SP1), dormancy in January (SP2), and pruning in March (SP3), and two-way ANOVA results.

Main Effect	Phenolic Acid				
	4-Hydroxybenzoic Acid	Caffeic Acid	Vanillic Acid	Ferulic Acid	Verbascoside
**Cultivar (Cv.)**					
‘Bova’	0.8 ± 0 ^b^	1.8 ± 0.2 ^bc^	0.8 ± 0 ^b^	0.6 ± 0.1	339 ± 66 ^ab^
‘Buža muška’	0.7 ± 0 ^b^	1.6 ± 0.3 ^c^	0.8 ± 0 ^b^	0.9 ± 0.1	233 ± 55 ^b^
‘Buža puntoža’	0.8 ± 0 ^b^	4.3 ± 0.6 ^a^	0.8 ± 0 ^b^	0.8 ± 0.2	438 ± 88 ^a^
‘Istarska bjelica’	1.3 ± 0.2 ^a^	1.3 ± 0.2 ^c^	1.0 ± 0.1 ^b^	0.9 ± 0.1	454 ± 117 ^a^
‘Leccino’	0.9 ± 0 ^b^	2.4 ± 0.3 ^b^	0.8 ± 0 ^b^	0.9 ± 0.1	364 ± 67 ^ab^
‘Rošinjola’	1.4 ± 0.3 ^a^	1.2 ± 0.1 ^c^	2.9 ± 0.6 ^a^	0.9 ± 0.2	482 ± 121 ^a^
**Sampling period (SP)**					
SP1	1.3 ± 0.2 ^a^	2.4 ± 0.3 ^a^	1.5 ± 0.4 ^a^	1.0 ± 0.1	108 ± 19 ^c^
SP2	0.8 ± 0 ^b^	2.6 ± 0.4 ^a^	1.2 ± 0.2 ^b^	0.8 ± 0.1	406 ± 41 ^b^
SP3	0.8 ± 0 ^b^	1.3 ± 0.2 ^b^	0.8 ± 0 ^c^	0.7 ± 0.1	639 ± 47 ^a^
**Cv.**	***	***	***	ns	**
**SP**	***	***	***	ns	***
**Cv. × SP**	***	*	***	**	*

Shown are means ± standard errors (n = 3). Different letters in a column represent statistically significant differences between mean values for each main effect at *p* ≤ 0.05 obtained by a two-way ANOVA and Tukey’s post hoc test (ns, not significant; *, *p* ≤ 0.05; **, *p* ≤ 0.01; ***, *p* ≤ 0.001).

**Table 3:** Concentrations (mg 100 g^−1^ dry matter) of selected flavonoids in leaves of six olive cultivars (Cv.), collected at three different sampling periods (SPs), harvesting in October (SP1), dormancy in January (SP2), and pruning in March (SP3), and two-way ANOVA results.

Main Effect	Flavonoids					
	Catechin	Luteolin-7-*O*- Glucoside	Luteolin	Rutin	Apigenin-7-*O*- Glucoside	Apigenin
**Cultivar (Cv.)**						
‘Bova’	42 ± 2 ^b^	374 ± 10 ^ab^	8.9 ± 1.1 ^c^	52 ± 2 ^ab^	23.6 ± 4.87 ^d^	0.8 ± 0.1 ^d^
‘Buža muška’	31 ± 1 ^c^	351 ± 18 ^b^	4.6 ± 0.7 ^d^	38 ± 3 ^c^	31.5 ± 9.46 ^c^	1.7 ± 0.3 ^c^
‘Buža puntoža’	53 ± 3 ^a^	422 ± 15 ^a^	5.2 ± 0.6 ^d^	32 ± 3 ^c^	45.5 ± 5.64 ^b^	0.4 ± 0.1 ^d^
‘Istarska bjelica’	26 ± 4 ^d^	199 ± 10 ^c^	10.6 ± 3.2 ^c^	50 ± 3 ^ab^	32.9 ± 5.46 ^c^	4.4 ± 0.9 ^a^
‘Leccino’	33 ± 2 ^c^	307 ± 31 ^b^	13.9 ± 1.8 ^b^	63 ± 12 ^a^	94.1 ± 16.2 ^a^	3.7 ± 0.7 ^ab^
‘Rošinjola’	34 ± 5 c	373 ± 27 ^ab^	28.9 ± 6.7 ^a^	36 ± 5 ^c^	42.9 ± 11.5 ^b^	4.0 ± 1.5 ^b^
**Sampling period (SP)**						
SP1	27 ± 3 ^b^	320 ± 24	19.8 ± 4.1 ^a^	37.8 ± 14.2 ^b^	45 ± 5	4.3 ± 0.9 ^a^
SP2	41 ± 2 ^a^	338 ± 19	8.2 ± 1.6 ^b^	51.9 ± 20.8 ^a^	45 ± 71	1.8 ± 0.3 ^b^
SP3	41 ± 2 ^a^	355 ± 22	8.1± 1.0 ^b^	45.8 ± 21.9 ^ab^	45 ± 6	1.4 ± 0.2 ^b^
**Cv.**	***	***	***	***	***	***
**SP**	***	ns	***	*	ns	***
**Cv. × SP**	***	**	***	ns	***	***

Different letters in a column represent statistically significant differences between mean values for each main effect at *p* ≤ 0.05 obtained by a two-way ANOVA and Tukey’s post hoc test (ns, not significant; *, *p* ≤ 0.05; **, *p* ≤ 0.01; ***, *p* ≤ 0.001).

**Table 4:** Concentrations of macronutrients (g kg^−1^ dry matter) and micronutrients (mg kg^−1^ dry matter) in leaves of six olive cultivars (Cv.), collected at three different sampling periods (SP), harvesting in October (SP1), dormancy in January (SP2), and pruning in March (SP3), and two-way ANOVA results.

Main Effect	Macronutrients					Micronutrients				
	P	K	Ca	Mg	Na	Fe	Zn	Mn	Cu	B
**Cultivar (Cv.)**										
‘Bova’	1.5 ± 0.0 ^c^	5.3 ± 0.4 ^d^	23 ± 1 ^a^	1.5 ± 0.1 ^bc^	0.4 ± 0.0	84 ± 5 ^ab^	22 ± 0 ^b^	32 ± 3 ^ab^	88 ± 20	20 ± 1 ^cd^
‘Buža muška’	1.1 ± 0.0 ^b^	7.2 ± 0.4 ^ab^	14 ± 1 ^c^	1.2 ± 0.1 ^cd^	0.5 ± 0.0	87 ± 4 ^a^	24 ± 1 ^ab^	30 ± 2 ^ab^	80 ± 16	23 ± 1 ^ab^
‘Buža puntoža’	2.5 ± 0.1 ^a^	8.0 ± 0.7 ^a^	12 ± 1 ^c^	1.0 ± 0.0 ^d^	0.4 ± 0.0	83 ± 6 ^ab^	27 ± 1 ^a^	28 ± 1 ^b^	78 ± 18	26 ± 1 ^a^
‘Istarska bjelica’	1.5 ± 0.1 ^c^	6.1 ± 0.6 ^c^	18 ± 1 ^b^	1.6 ± 0.1 ^b^	0.4 ± 0.1	79 ± 5 ^ab^	22 ± 1 ^b^	27 ± 2 ^b^	64 ± 10	22 ± 1 ^bc^
‘Leccino’	1.9 ± 0.1 ^b^	6.8 ± 0.5 ^bc^	22 ± 1 ^a^	1.1 ± 0.1 ^d^	0.5 ± 0.0	74 ± 5 ^b^	20 ± 1 ^b^	37 ± 2 ^a^	80 ± 15	19 ± 1 ^d^
‘Rošinjola’	1.6 ± 0.1 ^c^	5.5 ± 0.6 ^d^	24± 1 ^a^	2.1 ± 0.1 ^a^	0.4 ± 0.0	83 ± 5 ^ab^	26 ± 1 ^a^	39 ± 3 ^a^	57 ± 19	21 ± 1 ^bcd^
**Sampling period (SP)**										
SP1	2.0 ± 0.1 ^a^	7.9 ± 0.3 ^a^	20 ± 2	1.3 ± 0.1	0.5 ± 0.0 ^a^	96 ± 2 ^a^	22 ± 1 ^b^	35 ± 2 ^a^	23. ± 1 c	22 ± 1 ^b^
SP2	1.8 ± 0.1 ^b^	6.7 ± 0.3 ^b^	19 ± 1	1.5 ± 0.1	0.4 ± 0.0 ^b^	85 ± 2 ^b^	24 ± 1 ^a^	29 ± 2 ^b^	82 ± 5 b	21 ± 1 ^b^
SP3	1.6 ± 0.1 ^c^	4.7 ± 0.3 ^c^	18 ± 1	1.4 ± 0.1	0.4 ± 0.0 ^b^	65 ± 2 ^c^	24 ± 1 ^ab^	33 ± 2 ^ab^	118 ± 8 a	24 ± 1 ^a^
**Cv.**	***	***	***	***	*	**	***	***	ns	***
**SP**	***	***	ns	ns	***	***	**	*	***	***
**Cv. × SP**	ns	ns	ns	ns	ns	ns	ns	ns	**	**

Shown are means ± standard errors (n = 3). Different letters in a column represent statistically significant differences between mean values for each main effect at *p* ≤ 0.05 obtained by a two-way ANOVA and Tukey’s post hoc test (ns, not significant; *, *p* ≤ 0.05; **, *p* ≤ 0.01; ***, *p* ≤ 0.001).

**Table 5:** Correlations between measured phenolic compounds and selected macro- and micronutrients.

Phenolic Compounds/ Nutrients	P	K	Cu	B
Hydroxytyrosol	0.18	−0.13	0.44	0.01
Tyrosol	0.23	0.35	−0.24	−0.37
Vanillin	0.19	−0.14	0.45	0.14
Oleuropein	0.00	−0.30	**0.65** **	0.21
TPC	−0.15	**−0.51** *	**0.79** **	0.20
4-Hydroxybenzoic acid	0.00	0.18	−0.37	0.12
Caffeic acid	**0.84** **	**0.67** **	−0.26	0.07
Vanillic acid	−0.07	0.09	−0.32	0.06
Ferrulic acid	0.29	0.33	−0.28	0.10
Verbascoside	−0.36	**−0.75** **	**0.70** **	0.21
Catechin	0.26	−0.21	**0.49** *	0.21
Luteolin-7-*O*-glucoside	0.25	−0.04	0.17	0.18
Luteolin	−0.07	0.11	−0.42	−0.06
Rutin	−0.36	−0.25	0.27	−0.45
Apigenin-7-*O*-glucoside	0.24	0.15	−0.03	−0.32
Apigenin	−0.02	0.24	−0.41	−0.07

* Pearson correlation coefficients (r) are bolded only for moderate (absolute r = 0.40–0.50) to strong (absolute r > 0.50) significant correlations. Significance: *—*p* ≤ 0.05, **—*p* ≤ 0.01, results without asterisk are not significant. TPC—total phenols concentration.

## Data Availability

The data supporting our findings and analyses are included in the article itself. Readers can access this data by referring to the article.

## Supplementary Materials

The following supporting information can be downloaded at: https://www.mdpi.com/article/10.3390/plants14172789/s1, Table S1: Correlations between selected phenolic compounds and selected nutrients; Table S2; Results of the two-way analysis of variance for olive (*Olea europaea* L.) leaf phenolic and mineral concentrations (SS—Sum of Squares, MS—Mean Square; F-value, *p*-value).
