# Supplementary material for: Optimising Olive Leaf Phenolic Compounds: Cultivar and Temporal Interactions
Source: Plants (Basel). 2025 Sep 5;14(17):2789. doi: 10.3390/plants14172789 (PMC12430329; doi:10.3390/plants14172789)
Supplement: Supplementary file 1 [file plants-14-02789-s001.zip › plants-3810529-supplementary.pdf]

Table S1. Correlations between selected phenolic compounds and selected nutrients.

| Cultivar | Phenolic compounds/ Nutrients | P | K | B |
|---|---|---|---|---|
| **'Istarska bjelica'** | Verbascoside | **-0,82**** | **-0,80**** | 0,35 |
| | Oleuropein | -0,61 | -0,62 | 0,03 |
| | TPC | **-0,76**** | **-0,78**** | 0,22 |
| **'Leccino'** | Verbascoside | -0,26 | **-0,67*** | **0,79**** |
| | Oleuropein | -0,33 | -0,52 | 0,61 |
| | TPC | -0,26 | -0,44 | 0,55 |
| **'Rošinjola'** | Verbascoside | **-0,87**** | **-0,94***** | -0,54 |
| | Oleuropein | **-0,94***** | **-0,86**** | -0,61 |
| | TPC | **-0,83**** | **-0,69*** | -0,65 |
| **'Buža muška'** | Verbascoside | -0,01 | -0,66 | 0,13 |
| | Oleuropein | 0,59 | -0,26 | -0,1 |
| | TPC | 0,12 | -0,58 | -0,17 |
| **'Buža puntoža'** | Verbascoside | **-0,79**** | **-0,85**** | **0,70*** |
| | Oleuropein | **-0,84**** | **-0,72** | 0,62 |
| | TPC | **-0,76**** | -0,66 | 0,6 |
| **'Bova'** | Verbascoside | -0,62 | **-0,83**** | **0,75*** |
| | Oleuropein | -0,28 | -0,38 | -0,34 |
| | TPC | -0,66 | **-0,80**** | 0,27 |

* Pearson correlation coefficients (r) are bolded only for moderate (absolute r = 0.40–0.50) to strong (absolute r > 0.50) significant correlations. Significance: * – $p \leq 0,05$, ** – $p \leq 0,01$, *** – $p \leq 0,001$, results without asterisk are not significant. TPC – total phenols concentration.

Table S2. Results of the two-way analysis of variance for olive (*Olea europaea* L.) leaf phenolic and mineral concentrations (SS - Sum of Squares, MS - Mean Square; F-value, p-value). Transformed variables are marked in green (log) or blue colour ($1/\sqrt{x}$). Significant values for main factors (cultivar, sampling period, and their interactions, $p < 0.05$) are marked in red.

| Effect | | CULTIVAR | SAMPLING PERIOD | CULTIVAR * SAMPLING PERIOD |
|---|---|---|---|---|
| Degr. of Freedom | | 5 | 2 | 10 |
| Hydroxytyrosol | SS | 11581.61 | 5091.66 | 2461.89 |
| Hydroxytyrosol | MS | 2316.32 | 2545.83 | 246.19 |
| Hydroxytyrosol | F | 85.529 | 94.004 | 9.090 |
| Hydroxytyrosol | p | <0.000001 | <0.000001 | <0.000001 |
| Tyrosol | SS | 251.005 | 18.080 | 29.547 |
| Tyrosol | MS | 50.201 | 9.040 | 2.955 |
| Tyrosol | F | 18.843 | 3.393 | 1.109 |
| Tyrosol | p | <0.000001 | 0.044666 | 0.382103 |
| 4-hydroxybenzoic acid | SS | 3.28520 | 2.99563 | 6.57041 |
| 4-hydroxybenzoic acid | MS | 0.65704 | 1.49782 | 0.65704 |
| 4-hydroxybenzoic acid | F | 58.787 | 134.013 | 58.787 |
| 4-hydroxybenzoic acid | p | <0.000001 | <0.000001 | <0.000001 |
| Catechin | SS | 4327.62 | 2403.19 | 1111.92 |
| Catechin | MS | 865.52 | 1201.60 | 111.19 |
| Catechin | F | 79.192 | 109.941 | 10.174 |
| Catechin | p | <0.000001 | <0.000001 | <0.000001 |
| Vanillic acid | SS | 30.97544 | 4.75257 | 18.38704 |
| Vanillic acid | MS | 6.19509 | 2.37628 | 1.83870 |
| Vanillic acid | F | 84.710 | 32.493 | 25.142 |
| Vanillic acid | p | <0.000001 | <0.000001 | <0.000001 |
| Caffeic acid | SS | 0.84060 | 0.32192 | 0.16376 |
| Caffeic acid | MS | 0.16812 | 0.16096 | 0.01638 |
| Caffeic acid | F | 28.608 | 27.390 | 2.787 |
| Caffeic acid | p | <0.000001 | <0.000001 | 0.011652 |
| Vanillin | SS | 152.4266 | 18.2294 | 6.0019 |
| Vanillin | MS | 30.4853 | 9.1147 | 0.6002 |
| Vanillin | F | 101.373 | 30.309 | 1.996 |
| Vanillin | p | <0.000001 | <0.000001 | 0.063342 |
| Ferrulic acid | SS | 0.88567 | 0.63593 | 4.04208 |
| Ferrulic acid | MS | 0.17713 | 0.31797 | 0.40421 |
| Ferrulic acid | F | 1.3607 | 2.4426 | 3.1051 |
| Ferrulic acid | p | 0.261956 | 0.101217 | 0.005972 |
| Verbascoside | SS | 382609 | 2549370 | 390059 |
| Verbascoside | MS | 76522 | 1274685 | 39006 |
| Verbascoside | F | 5.1819 | 86.3196 | 2.6414 |
| Verbascoside | p | 0.001106 | <0.000001 | 0.015860 |
| Luteolin-7-*O*-glucoside | SS | 269068 | 10901 | 79171 |
| Luteolin-7-*O*-glucoside | MS | 53814 | 5451 | 7917 |
| Luteolin-7-*O*-glucoside | F | 22.554 | 2.284 | 3.318 |
| Luteolin-7-*O*-glucoside | p | <0.000001 | 0.116416 | 0.003848 |
| Rutin | SS | 6411.5 | 1809.8 | 3934.5 |
| Rutin | MS | 1282.3 | 904.9 | 393.5 |
| Rutin | F | 5.3545 | 3.7787 | 1.6430 |
| Rutin | p | 0.000884 | 0.032383 | 0.133771 |
| **Apigenin-7-*O*-glucoside** | SS | 1.8553 | 0.0026 | 0.2445 |
| **Apigenin-7-*O*-glucoside** | MS | 0.3711 | 0.0013 | 0.0245 |
| **Apigenin-7-*O*-glucoside** | F | 88.08 | 0.31 | 5.81 |
| **Apigenin-7-*O*-glucoside** | p | <0.000001 | 0.733682 | 0.000038 |
| Oleuropein | SS | 1.343521E+08 | 2.113721E+08 | 6.114735E+07 |
| Oleuropein | MS | 2.687043E+07 | 1.056861E+08 | 6.114735E+06 |
| Oleuropein | F | 45.653 | 179.561 | 10.389 |
| Oleuropein | p | <0.000001 | <0.000001 | <0.000001 |
| Luteolin | SS | 2.76339 | 1.17540 | 0.57959 |

| Effect | | CULTIVAR | SAMPLING PERIOD | CULTIVAR * SAMPLING PERIOD |
|---|---|---|---|---|
| Degr. of Freedom | | 5 | 2 | 10 |
| Luteolin | MS | 0.55268 | 0.58770 | 0.05796 |
| Luteolin | F | 50.390 | 53.583 | 5.284 |
| Luteolin | p | <0.000001 | <0.000001 | 0.000092 |
| Apigenin | SS | 2.02386 | 0.60895 | 1.18627 |
| Apigenin | MS | 0.40477 | 0.30447 | 0.11863 |
| Apigenin | F | 53.733 | 40.419 | 15.748 |
| Apigenin | p | <0.000001 | <0.000001 | <0.000001 |
| TPC | SS | 3.641104E+06 | 3.414378E+07 | 2.733698E+06 |
| TPC | MS | 7.282209E+05 | 1.707189E+07 | 2.733698E+05 |
| TPC | F | 5.039 | 118.140 | 1.892 |
| TPC | p | 0.001334 | <0.000001 | 0.079122 |
| P | SS | 7.2571 | 1.4066 | 0.4508 |
| P | MS | 1.4514 | 0.7033 | 0.0451 |
| P | F | 66.428 | 32.190 | 2.063 |
| P | p | <0.000001 | <0.000001 | 0.054809 |
| K | SS | 49.364 | 95.421 | 9.201 |
| K | MS | 9.873 | 47.710 | 0.920 |
| K | F | 19.727 | 95.332 | 1.838 |
| K | p | <0.000001 | <0.000001 | 0.088632 |
| Ca | SS | 0.64994 | 0.02621 | 0.04972 |
| Ca | MS | 0.12999 | 0.01310 | 0.00497 |
| Ca | F | 32.00 | 3.23 | 1.22 |
| Ca | p | <0.000001 | 0.051420 | 0.309158 |
| Mg | SS | 7.0651 | 0.2463 | 0.3510 |
| Mg | MS | 1.4130 | 0.1231 | 0.0351 |
| Mg | F | 28.626 | 2.495 | 0.711 |
| Mg | p | <0.000001 | 0.096683 | 0.708194 |
| Fe | SS | 912.3 | 8689.6 | 543.6 |
| Fe | MS | 182.5 | 4344.8 | 54.4 |
| Fe | F | 3.591 | 85.515 | 1.070 |
| Fe | p | 0.009764 | <0.000001 | 0.409491 |
| Zn | SS | 276.80 | 47.58 | 96.08 |
| Zn | MS | 55.36 | 23.79 | 9.61 |
| Zn | F | 10.073 | 4.329 | 1.748 |
| Zn | p | 0.000004 | 0.020664 | 0.107266 |
| Mn | SS | 1199.52 | 402.03 | 399.01 |
| Mn | MS | 239.90 | 201.01 | 39.90 |
| Mn | F | 5.685 | 4.763 | 0.945 |
| Mn | p | 0.000580 | 0.014613 | 0.505153 |
| Cu | SS | 0.0928 | 4.7362 | 0.3358 |
| Cu | MS | 0.0186 | 2.3681 | 0.0336 |
| Cu | F | 2.35 | 300.06 | 4.26 |
| Cu | p | 0.060354 | <0.000001 | 0.000602 |
| B | SS | 243.33 | 84.09 | 124.13 |
| B | MS | 48.67 | 42.04 | 12.41 |
| B | F | 12.393 | 10.706 | 3.161 |
| B | p | <0.000001 | 0.000225 | 0.005319 |
| Na | SS | 0.3212 | 1.0738 | 0.2542 |
| Na | MS | 0.0642 | 0.5369 | 0.0254 |
| Na | F | 2.277 | 19.030 | 0.901 |
| Na | p | 0.067464 | 0.000002 | 0.542021 |
